# Risk factor analysis for adverse prognosis of the fetal ventricular septal defect (VSD)

**DOI:** 10.1186/s12884-023-05969-9

**Published:** 2023-09-21

**Authors:** Wang Shan, Xia Yuanqing, Zhu Jing, Wu Xi, Guo Huifeng, Wu Yi

**Affiliations:** 1 Prenatal Diagnostic Center, International Peace Maternity & Child Health Hospital, Shanghai Jiao Tong University, School of Medicine, Shanghai, China; 2 Bio-X Institutes, Key Laboratory for the Genetics of Developmental and Neuropsychiatric Disorders (Ministry of Education), Shanghai Jiao Tong University, Shanghai, China; 3 Department of Gerontology, Shanghai Jiao Tong University Affiliated Sixth People’s Hospital, Shanghai, China; 4 Shanghai Key Laboratory of Embryo Original Disease, Shanghai, China

**Keywords:** Congenital heart disease, Ventricular septal defect, Prenatal diagnosis, Chromosome aberration, Defect size, Defect location, Fetal outcome

## Abstract

**Background:**

Ventricular septal defect (VSD) is the most common subtype of congenital heart disease. In the present study, we aimed to determine whether chromosome aberration was associated with the occurrence of VSD and evaluate the association of VSD size, location and chromosome aberration with adverse outcomes in the Chinese fetuses.

**Methods:**

Fetuses with VSD and comprehensive follow-up data were included and evaluated retrospectively. Medical records were used to collect epidemiological data and foetal outcomes. For VSD fetuses, conventional karyotype and microarray analysis were conducted. After adjusting confounding factors by using multivariable logistic regression analyses, the association between chromosome variations and VSD occurrence was explored. The association between defect size, location and chromosome aberrations and adverse foetal outcomes was also investigated.

**Results:**

Chromosome aberration was the risk factor for VSD occurrence, raising 6.5-fold chance of developing VSD. Chromosome aberration, peri-membranous site and large defect size of VSD were significant risk factors of adverse fetal outcome. Chromosome aberrations, including pathogenic copy number variations (CNVs) and variations of uncertain significance (VUS), were both risk factors, increasing the risk of the adverse fetal outcome by 55.9 times and 6.7 times, respectively. The peri-membranous site would increase 5.3-fold risk and defects larger than 5 mm would increase the 7.1-fold risk for poor fetal outcome.

**Conclusions:**

The current investigation revealed that chromosomal abnormalities, large defects, and the peri-membranous site were all risk factors for poor fetal outcomes. Our study also indicated that chromosome aberration was one of risk factors for the VSD occurrence.

**Supplementary Information:**

The online version contains supplementary material available at 10.1186/s12884-023-05969-9.

## Background

Congenital heart disease (CHD) is the most common congenital disability, affecting approximately 8 to 9 per 1000 live births [1, 2]. It is the leading cause of neonatal morbidity and mortality, fetal demise, and pregnancy termination. CHD has a broad clinical phenotypic spectrum. Ventricular septum defect (VSD) is the most common subtype [3]. It has been reported that the prevalence of VSD varied from 1.73–5% in liveborn neonates with regional differences [4, 5], and VSD was observed in 25–40% of children with CHDs [4, 6, 7]. Septal defects can be classified as peri membranous, muscular, sub arterial, or inflow, depending on where they originate in the interventricular septum. Echocardiography is the primary imaging modality for diagnosing and monitoring VSDs [8]. Children with a VSD risk contracting endocarditis, developing lung infections, developing ventricular arrhythmias, and passing away from heart failure or pulmonary hypertension [9]. VSD and atrial septal defect (ASD) risk factors may differ. For instance, maternal alcohol misuse, being overweight, and obesity are linked to VSDs but not ASDs [8]. On the other hand, the influence of maternal BMI is exclusively seen in ASDs [10]. High maternal age (≥ 35), which appears to affect both VSDs and ASDs, is one of the maternal features associated with the risk of septal heart defects (SHDs). Smoking, drug misuse, diabetes, and some diseases during pregnancy appear to be risk factors [11].

The outcome of patients with VSD depends on several factors, such as defect size and location, and whether it is combined with chromosomal abnormalities or whether it is combined with other structural anomalies. Several studies have evaluated the outcome of VSD patients. However, most of these data came from postnatal study cohorts [9, 10]. In recent years, improvements in ultrasound equipment and the widespread use of fetal echocardiography have led to an increase in the prenatal detection of VSD [11, 12]. There is currently a lack of information on patient outcomes throughout the perinatal period. Therefore, under the current medical care paradigm of improving the identification of prenatal VSD, it is of significant clinical value to examine the outcomes of VSD fetuses during the perinatal period.

As a result, the current study on VSD fetuses was designed to investigate the relationship between chromosomal abnormalities and VSD occurrence and determine whether there is a relationship between chromosomal abnormalities, VSD size or location, and adverse fetal outcomes.

## Methods

### Clinical data

This is a retrospective case-control study of fetuses with VSDs detected by fetal echocardiography conducted in our hospital from Jan 2019 to May 2021. Our center is a tertiary referral center with annual deliveries of approximately 10000 to 12000. Patients were referred to our center for a routine second-trimester anomaly scan. Fetuses with suspected CHD were offered fetal echocardiography for further screening. Once the CHD was diagnosed by fetal echo, an invasive procedure would be suggested to patients to detect the pathogenic chromosomal aberrations. A standard fetal anomaly scan control group conducted between Jan 2019 and May 2021 was assembled from pregnancies who accepted invasive procedures due to different clinical indications. The ethical committee of International Peace Maternity & Child Health Hospital approved this study.

The baseline information was collected from the computerized patient files, including maternal age, gravidity, parity, race, conception mode, and pre-pregnancy BMI (calculated as kg/height in m^2^). Pregnancy outcomes included gestation at delivery, delivery mode, Apgar score, birth weight, and birth height. The adverse fetal outcome included termination of pregnancy in the second trimester, premature delivery, fetal demise, neonatal death, and severe asphyxia of newborns. Regarding the defect size, fetuses were divided into three groups according to the defect sizes: small (< 3mm), mild (3 ~ 5mm), and large (≥ 5 mm). As for the defect location, fetuses were divided into three groups: muscular, membranous and outlet tract defects. Since fetuses with VSD and extra-cardiac anomalies would have higher rates of chromosomal abnormalities, fetuses with extra-cardiac malformations were excluded from the present cohort.

### Fetal echocardiography

Ultrasound equipment with a transabdominal 2-4-MHz curvilinear transducer, such as the Voluson E10 (GE Healthcare, Milwaukee, WI) or the iE33 (Phillips Medical Systems, Bothel, WE), was used by two professional sonographers in our facility to perform the foetal echocardiographic investigations. A detailed and complete echocardiographic examination was performed, which included biometric measurements along with a sequential scanning of each view: 4-chamber view, 3-vessel view, trachea and 3-vessel view, outflow tract view, and aortic and ductal arches view. Examining the interventricular septum was completed with Color Doppler imaging from at least two different planes. All the ultrasound assessments followed the guidelines of scanning and diagnosis of fetal cardiac disease [13–15].

### Chromosome testing

After signing the informed consent, amniotic fluid was collected to perform the fetal karyotype and microarray analysis. Amniotic fluid cells were cultured in two independent flasks. Karyotype analysis was performed following standard protocol using G-banding. Chromosomal microarray was performed following these procedures: First, amniotic fluid DNA was extracted using DNeasy Blood & Tissue Kit protocol(Qiagen, Germany): 5 ml amniotic fluid was centrifuged to remove the supernatant and get the precipitate at the bottom of the centrifuge tube. After adding digestive Buffer and 10µl proteinase K, mixed thoroughly and incubated at 56℃ for 10 minutes. 200µl ethanol was added and then pipetted the mixture into a DNeasy Mini spin column placed in collection tube. After centrifuging and washing for twice, we used a new tube with 200ul buffer AE to contain the flow-through and incubated at room temperature for 1 minute to dissolve DNA completely. Finally we centrifuged to elute the DNA.

Then Affymetrix CytoScan™ 750K Microarray Chips (Applied Biosystems™, ThermoFisher Scientific, USA) were used for array CGH studies following the standard method given by manufacturers; the chips were then scanned with GeneChip Scanner, and finally, we transferred scanned images to data using Chromosome Analysis Suite (ChAS, Applied Biosystems™, ThermoFisher Scientific, USA); All data were aligned to the Human Genome release 38 (hg38). Categorization of CNVs as benign, likely benign, Variants of uncertain significance (VUS), likely pathogenic or pathogenic was performed based on the American College of Medical Genetics (ACMG), and the Clinical Genome Resource (ClinGen) published ACMG TECHNICAL STANDARDS on Nov 06, 2019.

### Statistical analysis

The control group was selected through propensity score matching, in which the greedy nearest neighbor matching propensity score algorithm was applied. Propensity score was estimated by multivariable logistic regression model, in which maternal ethic, maternal age at delivery and maternal pre-pregnancy BMI were included. The proportion of case and control was set at 1:4 and matched them using caliper 0.1. R statistics software was utilized with Matchit software package.

The statistical description was made using percentages for categorical variables and mean and standard deviation for continuous variables. Where appropriate, the group difference was examined using the chi-squared test, t-test, or Mann–Whitney U test. Univariate and multivariable logistic regression analyses were used to determine the relationship between pathogenic CNVs and VSD. These analyses were also used to examine further the relationships between pathogenic CNVs, the location of VSD (muscle, peri-membrane, or outflow tract), and defect size with unfavorable fetal outcomes in the VSD group. In the multivariate model, maternal ethic, maternal age at delivery, maternal pre-pregnancy BMI, multiple births, and mode of conception were adjusted.

All the analyses were performed with the Statistical Package for the Social Sciences (SPSS) (IBM-SPSS Statistics v22.0, Inc Chicago, IL). A statistical significance level was set at a 2-tail *p*-value < 0.05.

## Results

### Epidemiological and clinical data of the included fetuses

**Fig. 1:**
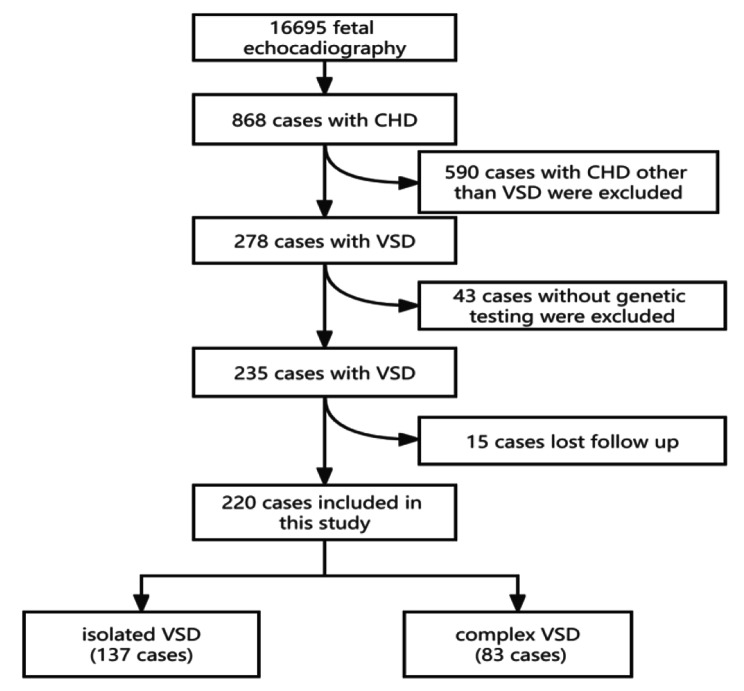
Flowchart of the enrolled fetuses

During the 3-year study period, 868 fetuses with various CHDs were detected among 16695 fetal echocardiographic examinations, presenting a CHD incidence of 5.2% in this referral-based study group. VSD was detected in 278 fetuses, presenting a prevalence of 32% among these CHD fetuses. In order to detect the underlying association between chromosomal aberrations and VSD, cases who refused invasive procedures and genetic testing (43 cases) were excluded from this cohort. Among the remaining 235 fetuses with VSDs, the pregnancy outcome could not be obtained in 15 cases after birth. Therefore, these 15 cases of loss to follow-up were excluded. Finally, 220 VSD cases of complete pregnancy outcomes were included in this study, with 137 cases of isolated VSD and 83 cases of VSD with other cardiac malformations. The flowchart of the fetus enrollment is shown in Fig. 1. A total of 1369 fetuses with normal cardiac structures were included in the control group, matched with gestational age at diagnosis. The clinical indications of control group for fetal echo and invasive procedure were as follows: advanced maternal age (746 cases), family history of CHD (21 cases), previous adverse pregnancy (210 cases), and fetal structural deformities other than CHD (392 cases).

Baseline information for control and VSD groups is shown in Table 1. There was a significant difference in maternal age between VSD and control groups. The control group was significantly older than the VSD group because women in the control group were predominantly at advanced maternal age requiring amniocentesis. A significant difference was observed in conception mode, with the VSD group showing a higher prevalence of natural conception than controls (*p* = 0.004). Chromosomal results and fetal outcomes were analyzed between the two groups, as shown in Table 2. The adverse fetal outcome was significantly higher in the VSD group than in the control group. The rates of pathogenic CNVs and termination of pregnancy (TOP) in the second trimester were significantly different, with higher pathogenic CNVs and higher TOP rates observed in the VSD group. A slight gender difference was observed, with female predominance in the VSD group. However, there was no significant difference concerning fetal gender when other demographic data were corrected. The birth weights and lengths between the two groups were also significantly different, with higher birth weights and higher birth lengths observed in control cases.

**Table 1:** Baseline characteristics of control and VSD group

	Control	VSD	*p*
Maternal age (years)	34.65 ± 4.51	29.81 ± 4.66	< 0.001
Primiparous (%)			< 0.001
*Y*	412 (46.82%)	70 (31.82%)	
Race			1.000
Chinese Han population	870 (99.54%)	218 (99.54%)	
Non-Han population	4 (0.46%)	1 (0.46%)	
BMI	21.82 ± 2.79	21.39 ± 2.8	0.247
< 18.5	71 (8.07%)	22 (10.19%)	
18.5 ~ 23.9	642 (72.95%)	163 (75.46%)	
24 ~ 27.9	138 (15.68%)	23 (10.65%)	
≥ 28	29 (3.3%)	8 (3.7%)	
Conception mode			0.004
Natural conception	751, 85.34%	204, 92.73%	
ART	129, 14.66%	16, 7.27%	
total	1369	220	

VSD: ventricular septal defect. BMI: body mass index. ART: assisted reproductive technology

**Table 2:** Comparative analysis of foetal outcome and CMA results in the control and VSD groups.

	Control	VSD	*p*
Fetal outcome			< 0.001
*Favourable*	857, 97.39%	126, 57.27%	
*Adverse*	23, 2.61%	94, 42.73%	
CMA results			< 0.001
*Normal*	839 _a_, 95.34%	178 _b_, 80.91%	
*Pathogenic*	23 _a_, 2.61%	29 _b_, 13.18%	
*VUS*	18 _a_, 2.05%	13 _b_, 5.91%	
TOP in the second trimester			< 0.001
*Y*	18, 2.05%	92, 41.82%	
Preterm birth			0.836
*Y*	76, 8.82%	12, 9.38%	
Fetal gender			0.023
*Male*	427, 48.52%	88, 40%	
*Female*	453, 51.48%	132, 60%	< 0.001
birth weight (g)	3280.15 ± 514.77	3096.84 ± 600.50	0.001
birth length (cm)	49.63 ± 2.16	48.83 ± 2.93	0.001
Total	1369	220	

*TOP: termination of pregnancy. VUS: variations of uncertain significance. VSD: ventricular septal defect

_a/b_:In pairwise comparisons between the three groups, the presence of the same letter indicates no significant difference between the two groups, whereas the absence of the same letter indicates a significant differen

### Association analysis of defect size and location with adverse fetal outcome

To investigate the association of defect size and location with the adverse fetal outcome, we divided VSD cases into three groups according to the defect size: small (< 3mm), mild (3-5mm), and large (≥ 5mm) (Table 3). The prevalence of adverse fetal outcomes was significantly different(*p* < 0.005), with the highest rate of adverse outcomes occurring in large groups (defects ≥ 5mm). Regarding the defect location, we divided VSD cases into three sub-groups: muscular, peri-membranous and outlet tract. Our results showed a significant difference in the average diameter of VSD among the three groups. The Peri-membranous VSD group had the largest average defect diameter (3.054 ± 1.2174mm). The muscular group had minor lesions (2.055 ± 0.6104mm). As expected, the incidence of adverse fetal outcomes in the peri-membranous group was significantly higher than in the muscular group (*p* = 0.000, Table 3).

**Table 3:** The association between defect size or location and adverse fetal outcome

	Number	VSD size	Combined with another cardiac anomaly	Pathogenic CNV	Adverse outcome
**VSD size**					
< 3	123, 55.91%	/	25_a_, 20.33%	18, 14.6%	37_a_, 30.1%
3 ~ 5	79, 35.91%	/	48_b_, 60.76%	8, 10.1%	43_b_, 54.4%
≥ 5	18, 8.18%	/	10_b_, 55.56%	3, 16.7%	14_b_, 77.8%
*p*			< 0.001	0.104	< 0.001
**VSD location**					
Muscular	33, 15.0%	2.055 ± 0.6104_a_	4, 12.1%_a_	2, 6.1%	4_a_, 12.1%
Perimemebranous	182, 82.7%	3.054 ± 1.2174_b_	76, 41.8%_b_	26, 14.2%	86_b_, 47.3%
Outlet tract	5, 2.3%	2.980 ± 0.5975_ab_	3, 60.0%_b_	1, 20.0%	4_b_, 80.0%
*p*		< 0.001	0.001	0.603	< 0.001

VSD: ventricular septal defect. CNV: copy number variation

_a/b_:In pairwise comparisons between the three groups, the presence of the same letter indicates no significant difference between the two groups, whereas the absence of the same letter indicates a significant difference

Among the 220 fetuses with VSDs, forty two cases were identified to have a chromosomal abnormality by CMA or katyotyping. The detailed information concerning the genetic testing of VSD group was shown in Supplement file 1. Univariate Regression Analysis was performed to analyze what factors are associated with VSD occurrence (results shown in Table 4). It was found that pathogenic CNVs, VUS and female fetuses were risk factors for VSD occurrence in the univariate regression model. Through a controlling procedure in the multivariate regression model, the significant findings of pathogenic CNVs(OR = 7.509, 95%CI: 3.740 ~ 15.075)and VUS (OR = 3.389, 95% CI: 1.491 ~ 7.702) remained (Table 4).

### Multivariate regression analysis on the association of chromosome aberrations, defect size and location between adverse fetal outcome

Further focusing on the adverse fetal outcome in the VSD group, it was found that pathogenic CNVs and VUS could increase the risk of the poor fetal outcome by 55.984 (OR = 56.984, 95%CI: 26.002 ~ 124.881) and 6.722 times (OR = 7.722, 95%CI: 3.322 ~ 17.948), respectively (Table 5). Compared to the location of VSD (muscular), peri membranous could increase the risk of poor fetal outcome(OR = 3.938, 95%CI: 1.265 ~ 12.264). Remarkably, the size of the defect was positively correlated with the risk of adverse fetal prognosis (OR = 1.746, 95%CI: 1.346–2.266). Mild (3 ~ 5mm) and large (≥ 5mm) defect sizes could increase the risk of poor fetal prognosis by 1.869 (OR = 2.869,95% CI:1.559 to 5.280) and 7.19 times (OR = 8.190, 95%CI:2.428 to 27.629) compared with small defect size (< 3mm).

**Table 4:** The risk factor of VSD occurrence

	*p*	OR(95%CI)	*p*	aOR(95%CI)^1^
CMA results				
Normal		1.000		1.000
Pathogenic	0.000	5.943 (3.359 ~ 10.516)	0.000	7.509(3.740 ~ 15.075)
VUS	0.001	5.943 (3.359 ~ 10.516)	0.004	3.389(1.491 ~ 7.702)
Fetal gender				
Male		1.000		1.000
Female	0.024	1.414 (1.047 ~ 1.909)	0.055	1.387 (0.993 ~ 1.937)

1 Obtained from the multivariable logistic regression model, with adjustment for maternal age, BMI, race, parity and mode of conception.

CMA:chromosomal microarray analysis. VUS: variations of uncertain significance

**Table 5:** The risk factor of the adverse fetal outcome in the VSD group

	*p*	OR(95%CI)	*p*	aOR(95%CI)^1^	*p*	aOR(95%CI)^2^
Chromosome results						
normal		1.000		1.000	/	/
pathogenic	0.000	54.590 (26.802 ~ 111.189)	0.000	56.984 (26.002 ~ 124.881)	/	/
VUS	0.000	8.055 (3.702 ~ 17.528)	0.000	7.722(3.322 ~ 17.948)	/	/
Location					/	/
muscular		1.000		1.000		1.000
perimemebranous	0.001	6.495(2.194 ~ 19.223)	0.001	6.351 (2.118 ~ 19.050)	0.018	3.938(1.265 ~ 12.264)
outlet tract	0.007	29.000 (2.558 ~ 328.713)	0.006	31.623 (2.654 ~ 376.740)	0.017	21.218(1.746 ~ 257.889)
Width	0.000	1.739 (1.351 ~ 2.240)	0.000	1.746 (1.346 ~ 2.266)	0.001	1.567(1.196 ~ 2.053)
< 3		1.000		1.000		1.000
3 ~ 5	0.001	2.776 (1.544 ~ 4.993)	0.001	2.869 (1.559 ~ 5.280)	0.018	2.170(1.144 ~ 4.116)
≥ 5	0.000	8.135 (2.509 ~ 26.372)	0.001	8.190 (2.428 ~ 27.629)	0.004	6.281(1.824 ~ 21.628)

^1^ Obtained from the multivariable logistic regression model, maternal age, BMI, race, parity and mode of conception adjustment.

^2^ Obtained from the multivariable logistic regression model, with adjustment for maternal age, BMI, race, parity, mode of conception and VSD width/location.

VUS: variations of uncertain significance. VSD: ventricular septal defect.

## Discussion

Our findings in the current study suggested that chromosomal aberration was a distinct risk factor for the development of VSD. We discovered that chromosomal abnormalities could raise the 6.5-fold chance of developing VSD. Aneuploidies were the most prevalent chromosomal abnormalities, including six trisomies 21, five trisomy 18, one trisomy 13 and one case of Turner syndrome. Cai et al. and Donnelly et al. reported that the most common chromosomal abnormalities among VSD patients were trisomies, Turner syndrome and 22q11.2 microdeletion [16, 17]. Bellucco et al. supported the strong association between chromosome alterations and cardiac malformation, especially in VSD [18]. Although recent studies suggest that isolated muscular defects do not increase the risk of chromosomal abnormalities [5, 19, 20], membrane defects correlate [19]. Due to the limitation of prenatal ultrasound, we did not distinguish isolated and non-isolated VSDs in the present study. We found that the pathogenic CNVs were much higher in the VSD group than in the control (13.18% vs 2.61%, *p* < 0.001), with aneuploidies and 22q11.2 deletion being the most common genetic aberrations. Our results were consistent with the previously reported 20–40% rates of chromosomal abnormalities in VSD patients [7, 16, 21, 22].

According to our results, chromosome aberration was not only a risk factor for VSD occurrence but also a risk factor for poor prognosis of VSD fetuses. This is understandable because couples would choose to terminate the pregnancy due to chromosomal abnormalities. To our surprise, variations of uncertain significance (VUS), generally considered harmless, were also demonstrated to increase the risk of VSD occurrence by 2.3 times. That indicated VUS also could be the pathogenicity of VSD occurrence. However, up to now, little study has reported the association between VUS and VSD occurrence.

Furthermore, we observed high rate of VUS in VSD group with high rate of pregnancy termination(13 cases with VUS, among them, 10 chose to terminate pregnancies). Pregnant women may experience a negative psychological impact due to cardiac structural abnormalities. The presence of VUS might exacerbate psychological stress by increasing the uncertainty of prenatal prognosis. As a result, VUS results contributed to a high rate of pregnancy termination.

In the present study, our data implied that defect size was an independent risk factor for the adverse outcome of VSD fetuses. Compared with defects < 3mm, defects with 3-5mm increased the 1.1-fold risk of adverse fetal outcomes. The defects ≥ 5mm increased the 5.2-fold risk of adverse prognosis. The larger the defect was, the worse the fetal outcome. According to Table 3, defect size did not increase the risk for pathogenic CNVs (*p* = 0.104). However, compared with minor defects, VSDs ≥ 3mm were at an increased risk for combination with other cardiac anomalies. VSDs combined with other cardiac defects, such as double outlet of the right ventricle or aorta coarctation, will increase the possibility of postanal surgical interventions. Due to the concerns of surgical risk and fetal outcome, some parents would choose to terminate the pregnancy, which may lead to a high rate of adverse fetal outcomes. Another reason for the more significant defects increasing the risk of adverse fetal outcomes is that larger ones are not quickly closed spontaneously. Li et al. reported that minor defects have the highest rates of closing spontaneously (83%). However, in their study, only 30% of patients with large VSDs could have a spontaneous closure, which means more surgical intervention is needed [23]. Cho et al. also indicated that a minor defect is a good prognostic factor for natural closure in utero [24]. Significant defects, usually challenging to close spontaneously, might cause hemodynamic change and impaired nutritional status. Hemodynamic change, such as left to right shunt or left ventricular outflow tract obstruction, may lead to intrauterine growth retardation (IUGR) and low birth weight. Levy reported that low birth weight and IUGR were more common in children with cardiovascular disease, making infants more susceptible to disease or infections [25]. We hypothesized that VSD fetuses with minor defects which could spontaneously close, especially in utero, would have a better hemodynamic and nutritional status, which may lead to a better fetal outcome.

Both peri-membranous and outflow tracts could raise the likelihood of unfavourable results depending on where the lesion is located. However, the number of outflow tract problems was insufficient to produce reliable statistical findings. As a result, this part was not addressed in the current study. Our data showed that peri-membranous deficiencies predominated over muscle ones. This outcome was consistent with several earlier studies [10, 23, 26]. According to our results, the membranous location was an independent risk factor for the adverse prognosis of VSD fetuses. Compared with muscular defects, the multivariable logistic regression results showed that membranous defects increase the 5.3-fold risk of adverse fetal outcomes. Since the adverse fetal outcome caused by VSD location might be associated with defect size, VSD size was used as a re-adjusting factor. Results indicated that membranous defects still increase the 2.9-fold risk of adverse fetal outcomes after re-adjusting. The main causes of membranous defects leading to the poor prognosis might be the following. First, unlike muscular defects, membranous defects are not quickly closed spontaneously. Nir suggested that the membranous defect is "covered" by tricuspid valve tissue during the process of closure, which is much more complicated than a fibrous tissue plug padding in the closure of a muscular defect [27]. Some researchers also supposed that isolated muscular VSD should be considered a delayed normal process of cardiac development, most of which could close spontaneously during gestation or in the first two years of life [26]. However, a membranous defect should be viewed as a pathological symptom, which may need surgical interventions after birth. Second, the average diameter of membranous defects is statistically more considerable than those of muscular ones (3.054 ± 1.2174mm vs 2.055 ± 0.6104mm, respectively). This finding was in line with Li et al. They also found that the average defect diameter of peri-membranous defects was larger than muscular ones [26].

Additionally, Zhao et al. observed that substantial defects (< 4 mm) and peri membranous sites are risk factors for VSD that do not close spontaneously [28]. Although the spontaneous closure of VSDs was not the primary focus of our research, we did find that peri-membranous sites and severe abnormalities are factors that increase the probability of adverse outcomes for the developing fetus. Moreover, several researchers proposed that membrane deficiency may increase the likelihood of chromosomal abnormalities. According to Gomez et al., one patient with perimembranous VSD had a chromosomal abnormality, showing a rate of 3.1% of chromosomal aberration(1/31), compared to no chromosomal anomalies in isolated muscle defects [29]. Chromosomal abnormalities would raise the probability of adverse foetal outcomes.

Strength and limitation

To our knowledge, this is one of the largest prenatal studies on fetal VSDs. It provides experience for prenatal diagnosis and helps the evaluation for the prognosis of the fetuses with VSDs. The main limitation is that TOP was included in the adverse fetal outcome, which would lead to a statistical bias. Another limitation is that VSD group contained both isolated VSDs and non-isolated VSDs, which could lead to statistical inaccuracy. Further large-sample studies with more specialized grouping are warranted to evaluate the outcome of fetuses with VSDs.

## Conclusions

In conclusion, our findings showed that chromosomal aberration was a risk factor for developing VSD and a poor fetal outcome. Small abnormalities and a muscle site, in terms of size and location, were good indicators of the prognosis for fetal outcomes. VSD fetuses had a poor prognosis due to chromosomal abnormalities, large defect size and the peri-membranous site. These results further suggested that clinicians could utilize the data from this study to assess the prognosis of fetuses with VSD.

### Electronic Supplementary Material

Below is the link to the electronic supplementary material

Supplementary Material 1

## Data Availability

Data will be provided upon request from the corresponding author.
