# Supplementary material for: Risk factor analysis for adverse prognosis of the fetal ventricular septal defect (VSD)
Source: BMC Pregnancy Childbirth. 2023 Sep 21;23:683. doi: 10.1186/s12884-023-05969-9 (PMC10515257; doi:10.1186/s12884-023-05969-9)
Supplement: Supplementary file 1 — Supplementary Material 1 [file 12884_2023_5969_MOESM1_ESM.docx]

Supplement file 1: the chromosomal abnormalities detected CMA in VSD group

| **Case**  **number** | **Clinical phenotype** | **CMA results** | **nature** |
| --- | --- | --- | --- |
| 43 | VSD | arr[hg19] Xq28(148,490,374-148,884,123)x2 | VUS |
| 68 | VSD, persistent left superior vena cava | arr[hg19] 7q31.31(120,832,282-121,033,289)x1 VUS | VUS |
| 93 | Tetralogy of Fallot | arr[hg19] 10q22.3q23.1(80,599,904-82,606,732)x1 | VUS |
| 95 | Tetralogy of Fallot | arr[hg19] 16q24.2q24.3(87,939,405-89,472,806)x1 | P |
| 101 | Tetralogy of Fallot | arr[hg19] 1q21.1q21.2(145,829,473-147,814,497)x3 | P |
| 130 | VSD | arr[hg19] 22q11.21(18,648,855-21,800,471)x1 | P |
| 131 | VSD, dysplasia of the right heart | arr(21)X3 | P |
| 141 | VSD | arr[hg19] 22q11.21(18,919,477-21,800,471)x1 | P |
| 142 | VSD | arr[hg19] 1p21.2p21.1(102,113,040-103,121,405)x1 | VUS |
| 143 | VSD, polyhydramnios | arr[hg19] 5p15.33p14.2(113,576-24,348,812)x1 | P |
| 145 | VSD, holoprosencephaly | arr(21)X3 | P |
| 146 | VSD, polyhydramnios | 21q11.2q22.3(15,016,486-48,093,361)x3 | P |
| 147 | VSD, right aortic arch | 22q11.21(21,033,586-21,800,471)x1 | P |
| 150 | VSD | arr[hg19] 16p13.11(15,058,820_16,527,659)x3 | P |
| 151 | VSD | arr(X)X2, (Y)X1 | P |
| 154 | Tetralogy of Fallot | arr[hg19] 22q11.21(18,631,364-21,800,471)x1 | P |
| 156 | Tetralogy of Fallot | arr[hg19] 16p11.2(29,580,020-30,190,029)x1 | P |
| 162 | Tetralogy of Fallot | 18p11.32p11.22(136,227-8,888,418)x1, 18p11.22q23(8,899,356-74,721,952)x4, 18q23(74,727,743-78,013,728)x1 | P |
| 164 | Tetralogy of Fallot | arr[hg19] 11p11.2p11.12(48,520,423-50,589,224)x4 | VUS |
| 167 | Tetralogy of Fallot | arr(21)X3 | P |
| 168 | VSD, pericardial effusion, fetal edema | arr(18)X3 | P |
| 172 | VSD, dysplasia of the right heart | arr[hg19] 11p15.5p15.4(2,649,849-2,892,546)x3 | VUS |
| 174 | VSD, right aortic arch | arr[hg19] 22q11.21(20,921,342-21,459,713)x1 | VUS |
| 175 | VSD, dextrocardia | arr[hg19] 1p36.21p36.12(16,105,084-22,493,485)x1 | P |
| 179 | VSD, hydrocephalus, choroid cyst | arr(18)X3 | P |
| 182 | VSD, interrupted aortic arch | arr[hg19] 22q11.21(18,648,855-21,800,471)x1 | P |
| 187 | VSD, coarctation of aorta, hypoplasia of the left heart | arr(21)X3 | P |
| 192 | Tetralogy of Fallot | 22q11.21(18,648,855-21,800,471)x1 | P |
| 193 | VSD, persistent truncus arteriosus | arr(18)X3 | P |
| 197 | VSD, persistent truncus arteriosus | arr[hg19] 1q44(247,541,412-248,058,661)x3 | VUS |
| 198 | VSD, transposition of the great arteries | arr[hg19] 16p13.11(15,325,072-16,282,869)x3 | VUS |
| 202 | VSD, coarctation of aorta | arr(18)X3 | P |
| 203 | VSD, transposition of the great arteries | arr[hg19] 5p13.2(36,960,017-37,613,158)x3 | VUS |
| 205 | VSD, persistent truncus arteriosus | arr(21)X3 | P |
| 206 | VSD, dysplasia of the right heart | arr(21)X3 | P |
| 208 | VSD, coarctation of aorta, cleft lip | arr(13)X3 | P |
| 210 | VSD | arr(X)X1 | P |
| 211 | VSD, double outlet right ventricle | arr[hg19] 5q35.1q35.3(171,532,277_180,696,371)x3,15q11.2q13.3(22,770,421_32,915,723)x4 | P |
| 217 | VSD, single atrium, persistent truncus arteriosus | arr[hg19] 21p11.2(9,832,936-9,833,196)x3 | VUS |
| 218 | VSD, transposition of the great arteries, dextrocardia | arr[hg19] 4p15.2(26,252,617-26,809,787)x3 | VUS |
| 219 | VSD, double outlet right ventricle | arr[hg19] 3p14.1(65,925,237-66,789,821)x3 | VUS |
| 220 | VSD, transposition of the great arteries, gastrochisis | arr(18)X3 | P |
